# Case Report: A case of diffuse midline glioma, H3 K27-altered presenting with long-segment spinal cord lesions

**DOI:** 10.3389/fonc.2026.1658494

**Published:** 2026-03-03

**Authors:** Chengcheng Lu, Songke Lu, Rongrong Li, Rui Pang, Ruijiao Zhao, Yao Cui, Jianguo Zhang, Wei Li, Huiqin Liu

**Affiliations:** 1 Department of Neurology, People’s Hospital of Henan University (Henan Provincial People’s Hospital), Zhengzhou, China; 2 Department of Neurology, Henan Provincial People’s Hospital, Zhengzhou, China; 3 Department of Pathology, Henan Provincial People’s Hospital, Zhengzhou, China; 4 Department of Oncology, Henan Provincial People’s Hospital, Zhengzhou, China; 5 Department of Neurosurgery, Henan Provincial People’s Hospital, Zhengzhou, China

**Keywords:** demyelinating diseases of the central nervous system, differential diagnosis, H3K27M, long segment spinal cord lesion, pediatric-type diffuse high-grade gliomas

## Abstract

Diffuse midline glioma H3 K27-altered is a subtype of pediatric-type diffuse high-grade gliomas, characterized as an infiltrative high-grade glioma involving midline structures with H3 K27 mutation. This clinically rare entity poses diagnostic challenges in the early stages and carries an extremely poor prognosis. We report a case of diffuse midline glioma H3 K27-altered, primarily manifesting as spinal cord lesions, and detail its clinical characteristics to enhance understanding of this disease entity. A 21-year-old male presented with “progressive quadriparesis over 7 months.” Seven months prior, he had developed bilateral lower limb weakness (grade II) following a cold. MRI at a local hospital revealed abnormal signals at T2–T5, diagnosed as “myelitis.” The disease progressed with lesion expansion despite treatment with glucocorticoid pulse therapy and intravenous immunoglobulin. The patient visited our hospital 7 months after the onset of the disease. At that time, the patient exhibited grade I muscle strength in the right upper limb and grade 0 in all other limbs, with sensory impairment below the neck. MRI revealed abnormal signals in the medulla oblongata and thoracic spinal cord. The patient received high-dose glucocorticoid pulse therapy again, but the symptoms did not improve despite treatment. The definitive diagnosis of diffuse midline glioma H3 K27-altered was established through spinal cord biopsy. The patient was discharged from the hospital due to respiratory failure 12 days post-diagnosis.

## Introduction

Diffuse midline glioma H3 K27-altered is a primary central nervous system tumor characterized by midline infiltration and H3 K27 mutation, featuring H3K27me3 loss, and is frequently associated with H3K27M mutation, EZH inhibitory protein (EZHIP) overexpression, or EGFR mutation. Classified as CNS WHO grade IV (1), it predominantly occurs in children, with rare adult cases, typically arising in midline structures, including the brainstem, thalamus, and spinal cord (2). Clinical manifestations vary depending on the tumor location and size and may potentially include motor deficits, sensory disturbances, and urinary/bowel dysfunction. The nonspecific presentation necessitates differentiation from various spinal cord pathologies, significantly increasing the difficulty of diagnosis. This study reports an adult-onset case presenting with longitudinally extensive spinal cord lesions that showed progressive deterioration despite immunotherapy and was ultimately diagnosed as diffuse midline glioma H3 K27-altered through spinal cord biopsy.

## Case presentation

**Figure 1:**
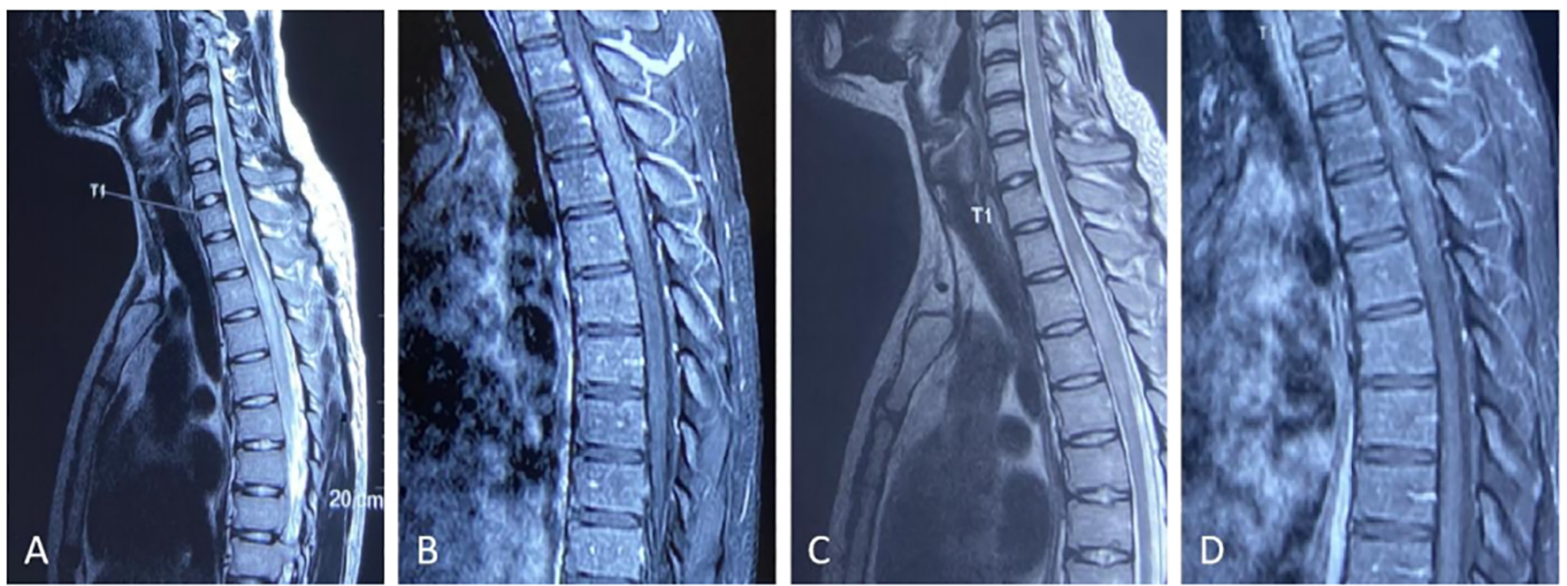
**(A, B)** Pre-treatment imaging prior to glucocorticoid pulse therapy. **(C, D)** Post-treatment imaging following glucocorticoid pulse therapy. Thoracic spine T2-weighted sagittal image **(A)** demonstrates abnormal signals at the T2–T5 levels. Sagittal T1-weighted contrast-enhanced sequence **(B)** reveals the lesion enhancement. The thoracic spine T2-weighted sagittal image **(C)** demonstrates persistent abnormal signals at T2–T5, while the sagittal T1-weighted contrast-enhanced sequence **(D)** shows lesion enhancement, both with reduced extent compared to pretreatment imaging.

**Figure 2:**
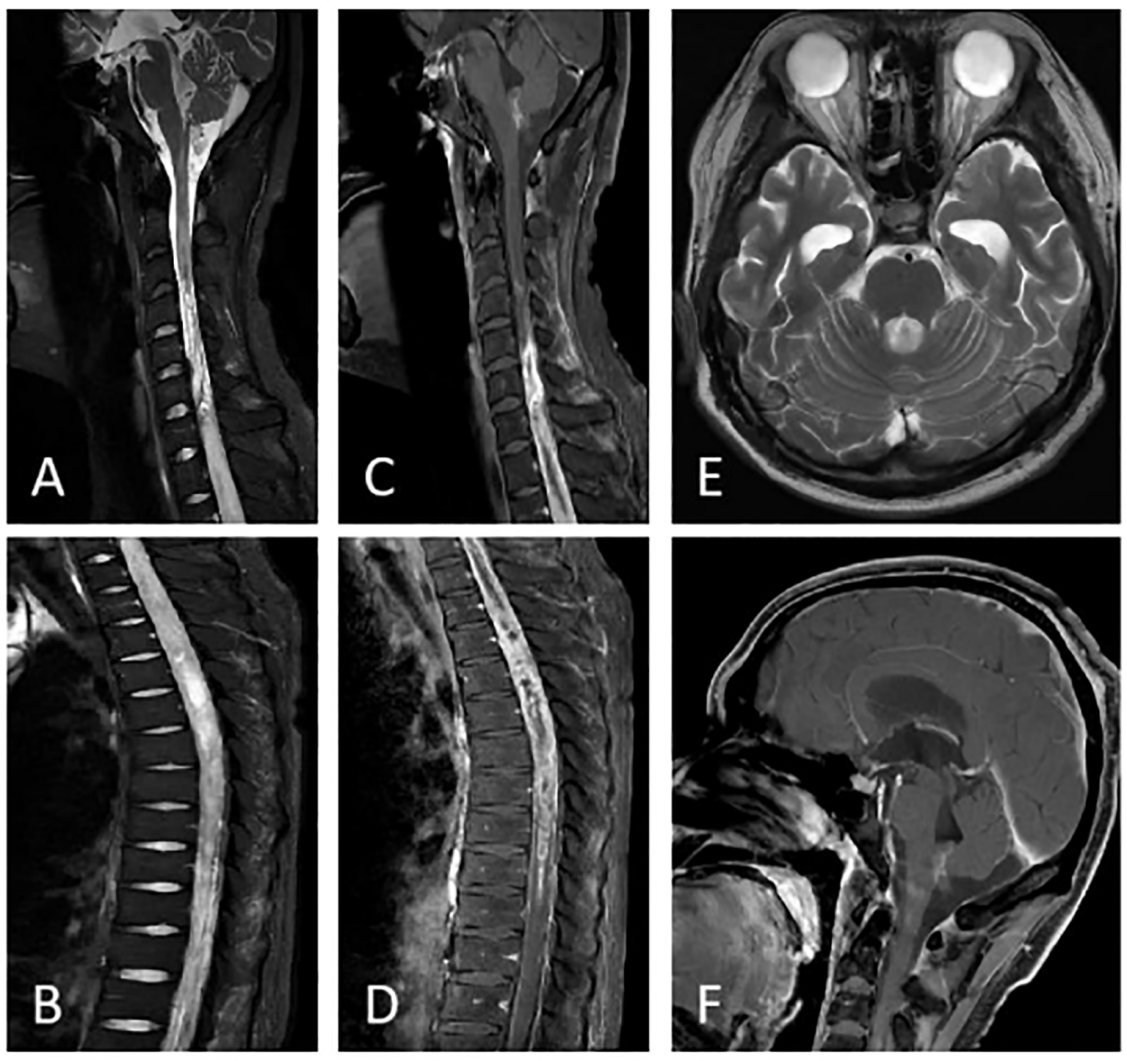
Post-glucocorticoid pulse therapy imaging at our hospital. On sagittal T2 **(A, B)** and T1 enhancement sequences **(C, D)**, the patient shows diffuse thickening of the spinal cord at the C4-L1 level, with uneven enhancement after contrast-enhanced scanning. Axial T2 **(E)** shows bilateral temporal pole expansion and patchy abnormal signal shadows in the fourth ventricle region; sagittal T1 enhanced scan **(F)** shows uneven enhanced signals in the fourth ventricle region and medulla oblongata.

Male patient, 21 years old, presented to the Neurology Department of Henan Provincial People’s Hospital on 24 November 2021 with the chief complaint of “progressive limb weakness for 7 months.” Seven months prior (April 2021), the patient developed left lower limb weakness following a cold that progressively worsened. Three days later, weakness developed in the right lower limb. He subsequently presented to the Department of Neurology of a local hospital. Neurological examination revealed grade II muscle strength in both lower limbs, and there were no abnormalities in deep and superficial sensations. The Expanded Disability Status Scale (EDSS) score was 8. MRI revealed abnormal spinal cord signals at the T2–T5 levels (**Figure 1**), and a clinical diagnosis of “myelitis” was considered. After receiving glucocorticoid pulse therapy, muscle strength recovered to grade IV in the left lower limb and grade III in the right lower limb, with the EDSS score dropping to 6 points. Follow-up MRI demonstrated reduced abnormal signals at the T2–T5 levels compared with the previous scan (**Figure 1**). The patient’s symptoms improved, and he was discharged. One week after discharge, the patient developed sudden sensory impairment below the T6 level, marked aggravation of weakness in both lower limbs, and urinary and bowel incontinence, prompting a visit to the local hospital. Neurological examination revealed grade 0 muscle strength in both lower limbs and sensory impairment below the T6 level, and the EDSS score increased to 8. Follow-up MRI demonstrated the extension of abnormal spinal cord signals to the T2–T8 levels. Cerebrospinal fluid (CSF) analysis revealed a white blood cell count of 15 × 10^6^/L ↑ (normal range: ≤8 × 10^6^/L) and a protein level of 1.6 g/L ↑ (normal range: 0.15 g/L–0.45 g/L) (**Figure 2**). After receiving glucocorticoid pulse therapy combined with intravenous immunoglobulin (IVIG) treatment again, the muscle strength of both lower limbs of the patient recovered to grade I, and the EDSS score remained at 8 points. Considering the stable condition, the patient requested discharge and was required to take oral glucocorticoid maintenance treatment at home. In the fourth month after the onset of the disease (August 2021), the patient stopped taking oral glucocorticoid drugs due to bone infarction in the right knee joint. As severe weakness in both lower limbs significantly impacted daily activities, the patient underwent rehabilitation therapy at a local hospital. Following rehabilitation, muscle strength in both lower limbs improved to grade II, but sensory impairment below the T6 level persisted, and the EDSS score remained at 8. Three days before visiting our hospital, the patient suddenly experienced weakness in her left upper limb. One day earlier, she experienced right upper limb weakness accompanied by hiccups. As his condition further deteriorated, he became uncertain about his underlying disease. Subsequently, he presented to the Department of Neurology at our hospital on 24 November 2021. He had been in good health in the past, and there was no similar disease history in his family.

Neurological examination on admission revealed the patient to be conscious and fluent in speech, with normal advanced cognitive function, no obvious abnormalities on cranial nerve examination, decreased muscle tone in all four limbs, muscle strength of grade I in the right upper limb, muscle strength of grade 0 in the remaining limbs, and disappearance of deep and superficial sensations below the neck. His EDSS score worsened to 9.0.Admission MRI revealed diffuse signal abnormalities in the cervical and thoracic spinal cord. Laboratory investigations revealed no significant abnormalities in the complete blood count, homocysteine level, thyroid function tests, rheumatologic panel, ANA/ENA autoantibodies, ANCA quantification, tumor markers, vascular endothelial growth factor (VEGF), and comprehensive immunological profile.

**Figure 3:**
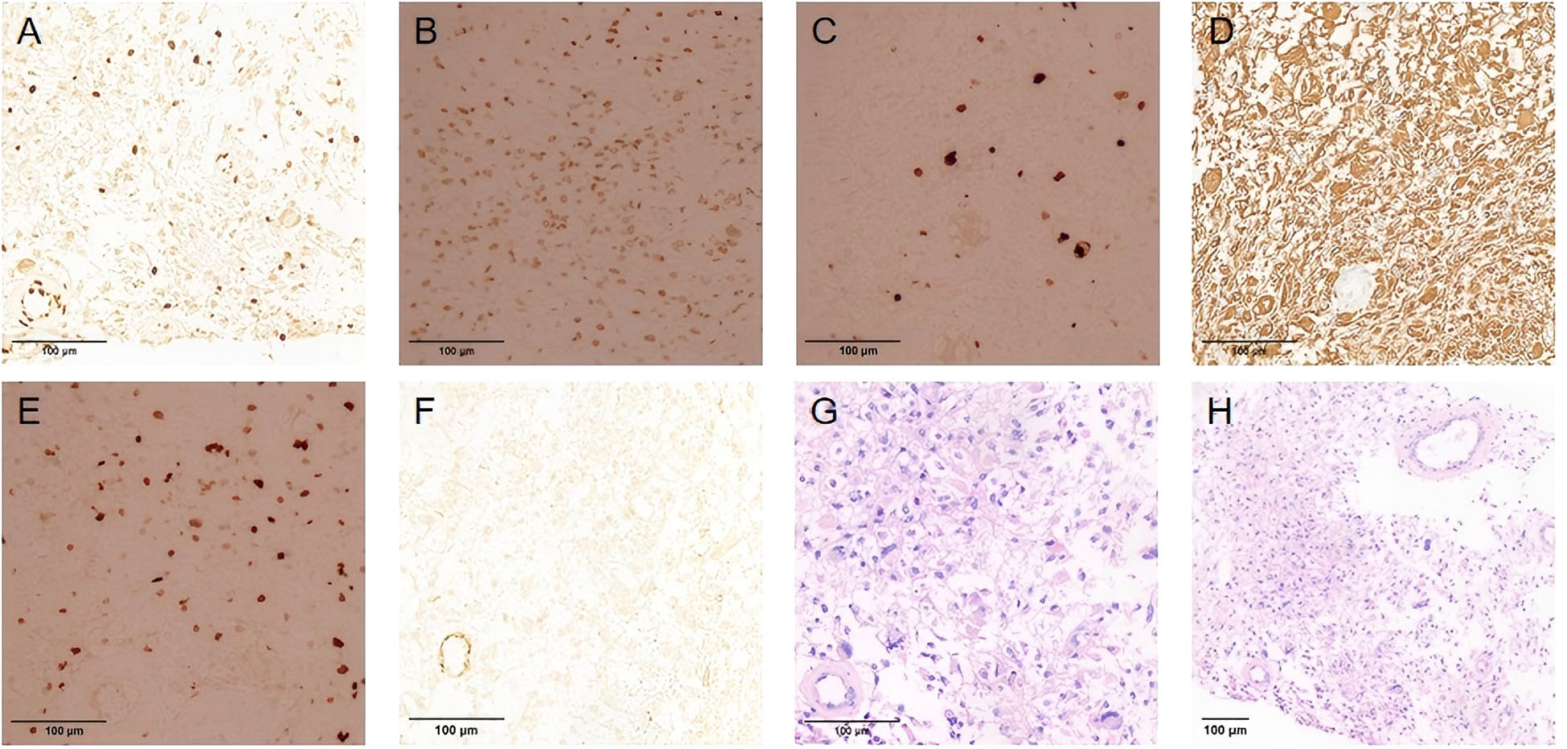
**(A)** Tumor cells show loss of H3K27me3 expression, indicating H3K27me3 mutation, with positive vascular endothelial cells serving as the internal controls. **(B)** Tumor cells showed positive H3K27M expression. **(C)** Partial positive expression of Ki67 in the tumor cells. **(D)** Positive GFAP expression in tumor cells. **(E)** Positive Olig-2 expression in tumor cells. **(F)** Negative expression of IDH1 in the tumor cells. **(G, H)** Histopathological sections of the submitted tissues showed a diffuse distribution of tumor cells and interstitial microvascular hyperplasia (hematoxylin and eosin staining). **(A–G)** Original magnification,×200; **(H)** Original magnification,×100.

**Figure 4:**
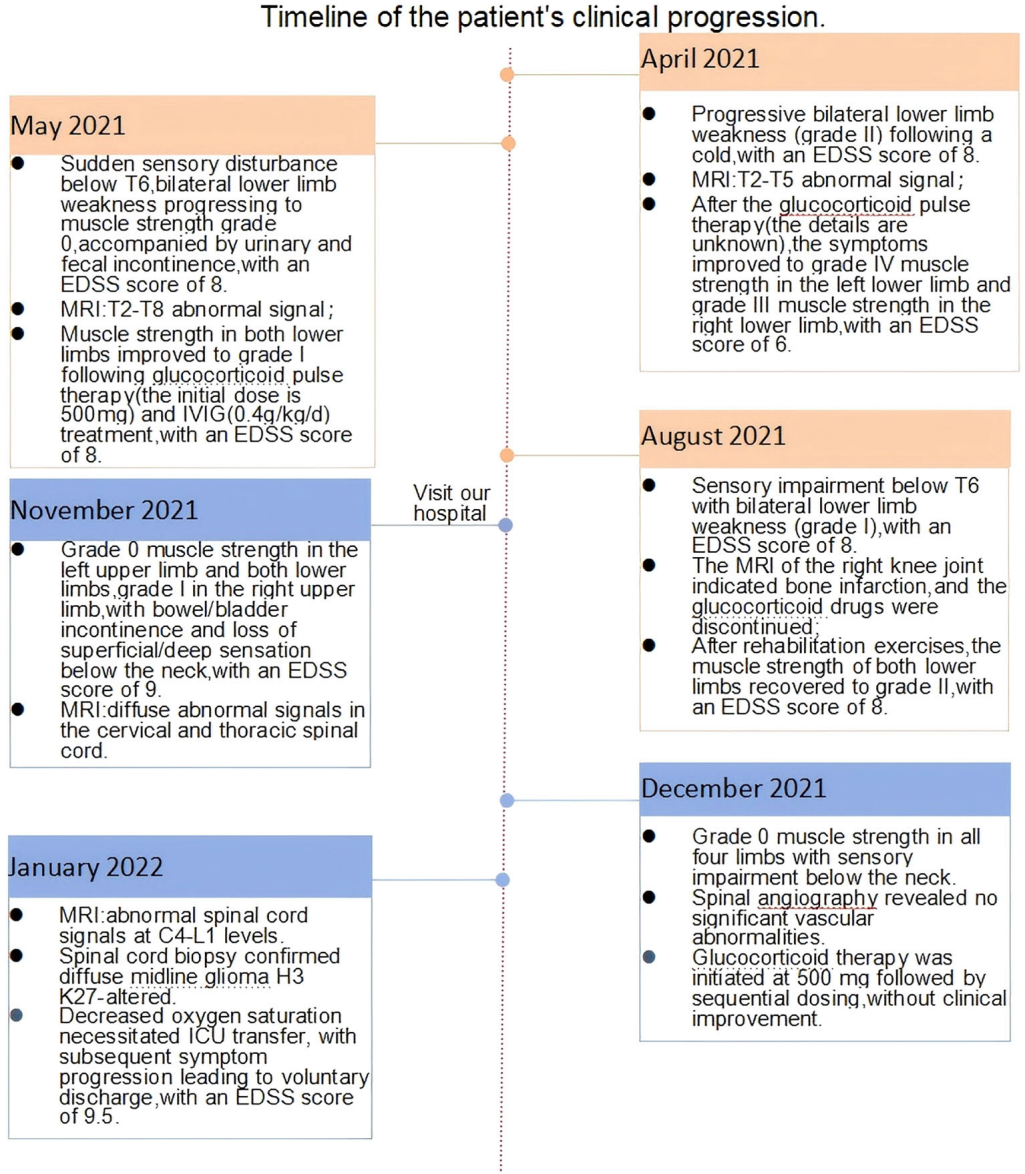
Timeline of clinical symptoms, imaging findings, diagnostic workup, treatment interventions, and EDSS score progression. This study utilized the muscle strength grading scale developed by the UK Medical Research Council (MRC) to assess the motor function of major joints in the participants. The MRC scale is a six-point scoring system (grades 0–V), defined as follows: Grade V: Full range of motion against maximum resistance. Grade IV: Full range of motion against moderate resistance, although weaker than the healthy side. Grade III: Full range of motion against gravity but unable to tolerate any additional resistance. Grade II: Full range of motion only with gravity elimination (e.g., horizontal plane movement).Grade I: Palpable muscle contraction without joint movement. Grade 0: No detectable muscle contractions.

During hospitalization, he suddenly developed slurred speech, impaired consciousness, delirium, and tachypnea. Head MRI revealed abnormal signals in the medulla oblongata and the fourth ventricle. However, his level of consciousness significantly improved the following day. Given the marked clinical fluctuations, spinal vascular pathology could not be ruled out, and he subsequently underwent “spinal angiography,” which showed no significant vascular abnormalities. Subsequently, he underwent a lumbar puncture, and the CSF WBC count was 5 ∗ 10^6^/L, with a protein level of 13.52 g/L↑. The CSF showed negative oligoclonal bands, and the serum aquaporin-4 (AQP-4), myelin oligodendrocyte glycoprotein (MOG), glial fibrillary acidic protein (GFAP), and myelin basic protein (MBP) antibodies were all negative. Preliminary considerations include the 1. Central nervous system immune-mediated myelitis and 2. Spinal cord tumor. He received high-dose methylprednisolone sodium succinate pulse therapy, but no clinical improvement was observed. Review of lumbar puncture, CSF WBC count: 5 ∗ 10^6^/L, protein:15.13 g/L↑. Follow-up MRI revealed abnormal changes in the spinal cord from the medulla to the level of the lumbar 1 (**Figure 2**). The disease progression in this patient was relatively rapid. Considering the imaging findings, cerebrospinal fluid characteristics, and clinical course, malignancy could not be ruled out. Therefore, positron emission tomography–computed tomography (PET-CT) was performed, which revealed spinal cord swelling from the medulla oblongata to the L1 level with heterogeneous density and progressively increased metabolism on dual-phase imaging. No evidence of malignancy was observed elsewhere in the body. The nature of the lesion remained undetermined, prompting a biopsy of the spinal cord. Findings during operation: (At the T5–4 level) The spinal cord was severely edematous with slight yellowish staining, showing diffuse, severe edema and high tension. The spinal cord was incised at the midline. A portion of the tissue was sent for pathological examination. Immunohistochemical staining of tumor cells: Olig-2 (partial+), GFAP (+), IDH1 (−), Ki67 (10%+), H3K27me3 (mostly−), H3K27M (partial+) (**Figure 3**). Molecular pathology results: N-MYC (FISH) (no amplification), IDH1/2 (no mutation), and TERT (no mutation). Pathological diagnosis: Diffuse glioma with focal necrosis, consistent with pediatric-type diffuse high-grade glioma, IDH-wildtype, not otherwise specified (NOS), CNS WHO grade 4 based on immunohistochemical markers and molecular testing. At this point, he was diagnosed with diffuse midline glioma H3 K27-altered. Subsequently, the patient exhibited a decreased spontaneous cough reflex and declining oxygen saturation, prompting transfer to the intensive care unit. The EDSS score increased to 9.5 points. The patient’s spontaneous cough reflex progressively deteriorated, ultimately leading to treatment withdrawal and voluntary discharge 12 days after diagnosis. The temporal profile of clinical and radiological features, diagnostics, therapies, and EDSS changes is shown in **Figure 4**.

## Results and follow-up

The patient eventually died a week after discharge.

## Discussion

Diffuse midline glioma H3 K27-altered is an infiltrative midline glioma. Studies indicate that patients with this tumor have a median overall survival of only 9.3 months, with a 2-year survival rate below 10%, demonstrating highly aggressive behavior and poor prognosis (3–5). This tumor typically occurs in the thalamus and spinal cord of children and adults (2). When a tumor occurs in the spinal cord, early clinical symptoms are often nonspecific. As the tumor enlarges, its mass effect may lead to neurological dysfunction, including sensory abnormalities, varying degrees of limb dysfunction, and bowel and bladder disturbances (6). These clinical manifestations significantly overlap with those caused by various spinal cord pathologies, resulting in diagnostic challenges during the initial evaluation and frequent misdiagnosis or missed diagnosis. Here, we report a case of an adult male with progressively worsening spinal cord lesions.

The patient’s symptoms began following a cold and rapidly progressed to weakness in the bilateral lower limbs. Thoracic spinal MRI revealed a longitudinally extensive spinal cord lesion that closely matched the typical features of acute myelitis, including preceding infection, acute onset, and predominant thoracic spinal cord involvement. Acute myelitis often results from post-infectious, autoimmune-mediated spinal cord injury. The clinical manifestations primarily include motor deficits, sensory abnormalities, and bowel/bladder dysfunction (7). The patient initially showed improvement in lower limb weakness following glucocorticoid administration, with follow-up thoracic MRI demonstrating a reduced lesion size, which further supported the preliminary diagnosis of myelitis. Glucocorticoids, with their anti-inflammatory, anti-edema, and immunosuppressive properties, are the primary treatment for various immune-mediated disorders (8). However, some patients with tumors may exhibit symptom relief following glucocorticoid administration due to reduced peritumoral edema. This nonspecific response can obscure clinical judgment, potentially leading to an initial misdiagnosis of inflammatory or autoimmune conditions, necessitating differentiation from demyelinating diseases.

Spinal MRI revealed lesions spanning more than three consecutive vertebral segments, consistent with the longitudinally extensive spinal cord involvement (≥3 vertebral segments) typical of neuromyelitis optica spectrum disorder (NMOSD) and myelin oligodendrocyte glycoprotein antibody-associated disease (MOGAD). However, the results of serum demyelinating antibody tests were negative. Although AQP-4 antibody-negative NMOSD exists, the patient lacked other core clinical features required by the 2015 diagnostic criteria for antibody-negative NMOSD (9).The patient demonstrated gradually progressive symptoms with spinal cord-restricted, continuous long-segment lesions that failed to meet the dissemination in time and space criteria, coupled with negative CSF oligoclonal bands, thus not fulfilling the 2017 McDonald diagnostic criteria for multiple sclerosis (MS) (10). Furthermore, studies have indicated that spinal cord cysts, necrosis, syrinx, and hemorrhage are more commonly associated with tumors and are generally uncommon in demyelinating diseases (11–13). Spinal MRI in this case demonstrated cystic degeneration, necrosis, and hemorrhage with involvement of both the spinal cord and meninges, suggesting a potential neoplastic etiology.

Inflammatory/autoimmune myelopathies may also occur secondary to systemic autoimmune diseases with CNS involvement, such as systemic lupus erythematosus and Sjögren’s syndrome, and typically present as longitudinally extensive spinal cord lesions (14). However, this patient’s negative serum ANA and anti-SSA/SSB antibody test results reduced the likelihood of an autoimmune disease.

The differential diagnosis of longitudinally extensive spinal cord lesions includes various etiologies, such as spinal vascular disorders, infectious diseases, and spinal neoplasms. Spinal vascular pathologies include spinal cord infarction and chronic vascular malformations. Spinal cord infarction typically presents with acute onset and T2 hyperintensity involving the anterior two-thirds of the spinal cord (i.e., “owl’s eye” or “snake eye” sign) (15). Spinal dural arteriovenous fistulas typically progress chronically, often affecting the central spinal cord and demonstrating flow voids on the cord surface. Spinal arteriovenous malformations refer to abnormal vascular clusters within or surrounding the spinal cord, supplied by single or multiple spinal arteries, with spinal angiography being the gold standard for diagnosis. However, the spinal angiogram of our patient showed no significant abnormalities.

Post-infectious or post-vaccination myelopathies may also present with longitudinally extensive spinal cord lesions. For instance, a minority of neurosyphilis and tuberculosis cases can demonstrate longitudinally extensive enhancing lesions with progressive and subacute clinical courses (16, 17). Additionally, during the coronavirus disease 2019 (COVID-19) pandemic, rare cases of myelitis following severe acute respiratory syndrome coronavirus 2 (SARS-CoV-2) infection or vaccination have been reported (18, 19). However, in this patient, the CSF white cell count showed only mild initial elevation followed by persistent normalization, with negative syphilis and tuberculosis screenings, negative CSF cultures, and negative SARS-CoV-2 nucleic acid testing results.

Spinal neoplastic disorders include primary intramedullary spinal cord tumors and metastatic lesions. Spinal cord metastases are rare, accounting for only 1%–3% of all intramedullary tumors. On MRI, metastases typically appear hypointense or isointense on T1-weighted imaging (T1WI) and hyperintense with heterogeneous signals on T2-weighted imaging (T2WI), demonstrating ring-like, patchy, nodular, or heterogeneous enhancement (20). However, the patient’s tumor markers and thoracic/abdominal/thyroid ultrasounds were normal, with no neoplasms detected on PET-CT. Ependymoma is the most common primary spinal cord tumor in adults. MRI features include a long-segment lesion with focal cord expansion and well-defined margins. Approximately 75% of cases exhibit homogeneous enhancement, while approximately 25% demonstrate hypointense bands at both ends of the tumor on T2WI (“cap sign”) (21). Astrocytomas demonstrate eccentric infiltrative growth with irregular margins, showing heterogeneous or absent enhancement on contrast imaging (22). Ependymomas and astrocytomas are the most common pathological types of spinal cord gliomas. When pathological morphology aligns with diffuse glioma and immunohistochemical/molecular testing reveals the H3 K27 mutation, the tumor is uniformly classified as WHO grade IV (23). These tumors most frequently arise in midline structures, including the brainstem, thalamus, and spinal cord, demonstrating high aggressiveness and extremely poor prognosis (24). Our patient exhibited diffuse glioma morphology in a midline location with H3K27M positivity on immunohistochemistry, ultimately supporting the diagnosis of diffuse midline glioma H3 K27-altered.

Although MRI and other imaging modalities provide crucial diagnostic clues for spinal cord tumors and PET-CT helps demonstrate hypermetabolism in tumor-associated spinal lesions, histopathological examination remains the gold standard for definitive diagnosis (25). However, in this case, spinal cord biopsy was performed only at the 8th month after symptom onset due to the invasive nature of the procedure, which carries risks of neurological deterioration, hemorrhage, infection, and cerebrospinal fluid leakage. This necessitates a careful clinical evaluation of its diagnostic value versus potential risks.

Reviewing the warning signs suggesting neoplasia in this case report, our patient exhibited continuous clinical progression over 6–7 months, which strongly indicated a tumorous etiology. In inflammatory/immune-mediated myelopathies, the time course from symptom onset to peak severity is typically shorter, presenting with acute or subacute progression. The patient exhibited progressive clinical deterioration and lesion expansion with persistent enhancement on contrast sequences despite high-dose glucocorticoid pulse therapy and intravenous immunoglobulin treatment, and later developed cystic degeneration, necrosis, and hemorrhage. During treatment, the patient’s CSF WBC count remained normal while protein levels showed persistent elevation (reaching several dozen times the normal values). In contrast, demyelinating spinal lesions typically exhibit normal or mildly elevated CSF protein levels. These findings strongly suggest a neoplastic etiology. Elevated protein levels may result from tumor-derived protein secretion, inflammatory exudation, and subarachnoid space obstruction. Despite immunotherapy, the persistent presence and continued growth of the tumor led to sustained protein elevation.

Compared with other cases diagnosed at an early stage, the patient in this case missed the potential therapeutic window. For instance, Peters et al. (26) reported a 39-year-old male who presented with progressive weakness in both upper limbs for 3–4 months. Prompt tumor resection was performed, and the patient was diagnosed with diffuse midline glioma H3 K27-altered. After undergoing tumor resection, radiotherapy, and chemotherapy, the patient’s survival time was extended to 31 months post-diagnosis. Yabuno et al. (27) reported two cases of patients diagnosed with diffuse midline glioma H3 K27-altered at an early stage, who survived for 14+ months and 22+ months respectively, and both were still alive at the time of the report’s publication. However, the patient in this case experienced continuous deterioration of self-function, a decline in quality of life, and lost the opportunity for any anti-tumor treatment after the final definitive diagnosis. This comparison strongly suggests that for “atypical inflammatory lesions” with poor therapeutic response, a more proactive strategy should be adopted to initiate histological evaluation as early as possible.

This study had certain limitations. As the patient rapidly developed severe complications, including hypoxemia, after diagnosis and was discharged automatically, we were unable to evaluate the potential response of this type of tumor to radiotherapy, chemotherapy, or any other palliative treatment. Furthermore, as this was a single case report, the generalizability of the findings and conclusions is limited.

## Conclusion

We report a rare case of an adult male presenting with longitudinally extensive spinal cord lesions that showed progressive deterioration despite immunotherapy and was ultimately diagnosed as diffuse midline glioma H3 K27-altered by spinal cord biopsy. This case reminds us that in patients with spinal cord lesions, the possibility of underlying tumors should be vigilantly considered when the following conditions occur: 1. Clinical symptoms continue to worsen despite prolonged and standardized immunotherapy; 2. Radiologically, the range of lesions gradually expands with persistent enhancement, and cystic changes, necrosis, or hemorrhage in the spinal cord may occur; 3. In terms of cerebrospinal fluid (CSF), the CSF protein level shows a persistent elevation without a significant increase in the CSF cell count. In such scenarios, invasive diagnostic methods, such as spinal cord biopsy, may need to be considered to improve early diagnosis and prevent missed diagnoses or misdiagnoses. Further research and exploration are needed for the treatment of this type of glioma.

## Data Availability

The datasets presented in this study can be found in online repositories. The names of the repository/repositories and accession number(s) can be found in the article/supplementary material.
